# Analysis of health literacy and health outcomes among farmers in border areas: an exploration based on a cross-sectional survey and structural equation modeling in South China

**DOI:** 10.3389/fpubh.2026.1757607

**Published:** 2026-03-16

**Authors:** Jiancheng Liang, Shichao Wu, Liping Huang, Nurul Hana Binti Zainal Baharin, Muhammad Fattah Fazel, Rusli Bin Nordin, Guiying Pan

**Affiliations:** 1 School of Public Health, Youjiang Medical University of Nationalities, Baise, Guangxi, China; 2 Faculty of Medicine, MAHSA University, Kuala Langat, Selangor, Malaysia; 3 Department of Public Health, Chongzuo Centre for Disease Control and Prevention, Chongzuo, Guangxi, China; 4 Department of Intensive Care Unit, Baise People's Hospital, Baise, Guangxi, China

**Keywords:** border areas, cross-sectional survey, farmers, health literacy, health outcomes, structural equation modeling

## Abstract

**Background:**

Farmers in border areas face unique health challenges, and this group’s health literacy and health outcomes are issues of social concern.

**Objective:**

The aim was to use the health literacy surveillance of residents in a prefecture-level city on the border of South China as the basic information to gain a comprehensive understanding of the current status of demographic characteristics, health literacy, and health outcomes of local farmers, and to explore the correlation among the three, to provide basic information and reference basis for related research and intervention.

**Methods:**

The study population was from Chongzuo City, Guangxi Zhuang Autonomous Region (*N* = 2,718). A questionnaire was completed using face-to-face interviews. The questionnaire included demographic characteristics, health literacy, and self-reported health status. The data were analyzed in a basic way, and the proposed structural equation model was fitted using AMOS.

**Results:**

The health literacy level of farmers in the border area of South China was 18.21, 48.38% self-reported their health as good, 18.87% suffered from chronic diseases, 7.62% suffered from accidental injuries in the past year, and median of out-of-pocket medical expenses in the past year = 300 RMB. the model fitted the data well. Demographic characteristics significantly affected health literacy and health outcomes (*p* < 0.001), with standardized path coefficients of −0.603 for health literacy and 0.682 for health outcomes. Health literacy also significantly affected health outcomes (*p* < 0.001), with a standardized path coefficient of 0.246.

**Conclusion:**

Farmers in the borderlands of South China have a low level of health literacy, a moderate level of health outcomes, and relatively low out-of-pocket medical expenses. Targeted health education or health promotion efforts can improve farmers’ health literacy and outcomes. Among them, middle-aged and older people, those with Separated/Divorced/Widowed marital status, low education level, and low income are the groups that need to be focused on.

## 1 Background

Border areas are located at the borders of countries and are usually geographically remote. Especially in developing countries, infrastructure is weak, and socio-economic health conditions are poor. In these areas, farmers, as the main occupational group, face many unique health challenges (1, 2). On the one hand, farmers have a low health literacy level due to the lack of adequate health education and health services, leading to greater difficulties in disease prevention and health behaviors (3). On the other hand, due to low-income levels and poor living conditions, farmers in border areas often lack adequate healthcare resources (4), resulting in higher rates of chronic diseases, hypertension, diabetes, unintentional injuries, and other health problems, and face problems with the availability of health services due to the uneven distribution of healthcare resources (5), which in turn affects their health outcomes (6).

Health literacy refers to an individual’s ability to obtain, understand, evaluate, and apply health information to make health decisions (7). Good health literacy is closely related to people’s health behaviors and can promote individual health behaviors, reduce disease incidence, and enhance health outcomes. Studies have shown that groups with higher health literacy perform better in preventive health behaviors, disease management, and quality of life (8, 9). For farmers in border areas, improving their health literacy helps increase their health awareness, improve health behaviors, and reduce the disease burden (10).

Studies on the relationship between health literacy and health outcomes have received increasing attention in recent years. Scholars at home and abroad have conducted many studies on health literacy behaviors and quality of life, among others (3). Although some explorations have taken the border area as the research territory, most have focused on issues such as insufficient health services and disease burden. On the other hand, although the health literacy survey and monitoring of Chinese residents has been conducted for 16 years (since 2008), the amount of research is relatively abundant (11). However, research on the association between health literacy and health outcomes among farmers in border regions remains limited, particularly when incorporating concurrent health outcome variables such as self-rated health, chronic diseases, accidental injuries, and out-of-pocket medical expenses. To address this gap, the present study draws on health literacy monitoring data from Chongzuo City, Guangxi Zhuang Autonomous Region, China. It aims to describe the current status of health literacy and health outcomes among local farmers and to identify their respective influencing factors. Building on the analysis results and a review of the literature, we then formulated hypotheses and constructed a structural equation model to examine the relationships among demographic characteristics, health literacy, and health outcomes in this border farming population. By doing so, this study fills the aforementioned literature gap and provides foundational data and a reference for future health education interventions and research targeting similar populations.

## 2 Research methodology

### 2.1 Research methods

In this study, residents whose occupation is farmer were screened from the health literacy monitoring database of residents in Chongzuo City, Guangxi, in 2022 as the research subjects. The health literacy monitoring of Chinese residents is organized by the National Health and Health Commission Publicity Department, with technical support provided by the China Health Education Center. Provincial health commissions were responsible for organizing and implementing the survey in their provinces (districts and cities), and the prefectural-level cities (districts and states) CDCs organized the counties (districts and county-level cities) to carry out the survey. Multi-stage stratified random sampling was used to identify survey respondents, and basic information, health literacy, and self-reporting of the study population’s health status were collected face-to-face. Personnel involved in the monitoring work were unified to receive professional training before the survey and questionnaires with crucial personal information and missing outcome variables were excluded from the data analysis. Information on the health literacy of Guangxi and national residents in the same period was obtained from the official website of the Chinese government (12, 13).

### 2.2 Survey sample and sampling

The resident population of Chongzuo City is 2.08 million, and the monitoring sample size was calculated as 3,267 according to the literature method (14). Three townships (streets) were randomly selected by Chongzuo CDC using the PPS method within each county (district and city) under its jurisdiction. Then, 3 neighborhood committees (villages) were sampled from each street (townships). Finally, 121 family households were randomly selected in each town (neighborhood committee), and 1 person from the resident population was randomly selected from each family household for the survey according to the KISH table method. Inclusion and exclusion criteria: (1) Permanent residents aged 15–69 years old who are not living collectively; (2) Regardless of whether or not they have a local household registration; (3) Permanent residents are those who have lived in the local area for a cumulative period of more than 6 months in the past 12 months. (4) Residents in groups, including schools, military bases, hospitals, prisons, nursing homes, dormitories, etc., were excluded.

In the end, 3,250 questionnaires were collected, with 3,242 valid questionnaires, an effective rate of 99.75%. Among them, those whose occupation is farmer accounted for 83.84% of the valid questionnaires, totaling 2,718, which is the sample of this study. The sample covers all 7 administrative areas (1 district, 1 city, and 5 counties) under the jurisdiction of Chongzuo City, involving 21 townships (streets) and 124 villages (neighborhood committees).

### 2.3 Questionnaire

The National Health Literacy Monitoring Questionnaire was supervised by the National Health Commission of the People’s Republic of China (NHC). The questionnaire consists of 71 questions on the respondents’ basic demographic characteristics, health literacy status, and self-reported health status. (1) Demographic characteristics include gender, age, ethnicity, marital status, family size, education level, occupation, whether or not they are of local household registration, annual household income (referred to as income), BMI (height and weight), whether or not they participate in regular physical exercise, whether or not they smoke, and whether or not they have heard of the “66 Articles of Chinese Citizens’ Health Literacy” (referred to as “HL66”). This section was used as a demographic variable in this study. (2) Self-reported health status included self-assessment of health, chronic disease, accidental injury in the past year, and out-of-pocket medical expenses in the past year. This component served as the health outcome variable in this study. (3) There were 56 questions on health literacy, including the three major health literacy dimensions of basic knowledge and concepts, lifestyles and behaviors, and basic skills, covering six categories of knowledge: scientific health concepts (HL01), infectious disease prevention and treatment (HL02), chronic disease prevention and treatment (HL03), safety and first aid (HL04), basic medical care (HL05), and health information (HL06). The resident was recognized as having health literacy with a score of 80% and above for total health literacy (HL00) or each health literacy category (three dimensions and HL01 - HL06). In this study, HL00 was used as a latent variable, HL01 - HL06 as observational variables of health literacy, and the three major dimensions were not explored. The questionnaire has been used since 2008 and has been revised and tested many times before and after, with an internal consistency reliability of 0.95 and a structural validity coefficient of 0.94 (14). The Cronbach’s *α* = 0.917, KMO = 0.922 for this questionnaire. In addition, because of the inconsistency of the measurement tools of health literacy in different countries, most of the data on the cross-sectional comparison of health literacy and related indicators in this study refer to the literature in mainland China.

### 2.4 Statistical analysis

Descriptive analysis and one-way analysis were conducted through SPSS 23.0 software. It mainly includes (1) sample characterization to describe the basic demographic characteristics of the sample. (2) Health literacy level situation and its influencing factors analysis. (3) Analysis of the status of health outcomes and their influencing factors. (4) Correlation analysis of demographic characteristics, health literacy, and health outcomes. Descriptive analysis was mainly based on the constituent ratio as an indicator, the chi-square test was used for single-factor analysis, and Spearman’s rank correlation was used for correlation analysis. The difference was considered to be statistically significant when *p* ≤ 0.05.

Literature review and analysis confirm that demographic characteristics are the strongest predictor of health literacy among multiple factors and also significantly shape health outcomes. Health literacy is therefore inferred to influence outcomes as well. Based on this, a structural model is proposed with three hypotheses: demographic characteristics directly affect health literacy; demographic characteristics directly or indirectly affect health outcomes; and health literacy directly affects health outcomes. The resulting initial model captures the relationships among three latent variables—demographic characteristics, health literacy, and health outcomes. The exogenous variables of demographic characteristics were age, literacy, marital status, annual household income, and “HL66”; the exogenous variables of health literacy (HL00) included the six categories of knowledge health literacy, HL01- HL06; and the health outcomes were measured by the four exogenous variables of self-assessment of health, chronic diseases, accidental injuries, and out-of-pocket medical expenses. Health outcomes are measured using four observable variables: self-rated health, chronic diseases, accidental injuries, and out-of-pocket medical expenses, as synthesized from the literature (15, 16). Model estimation was conducted using the maximum likelihood (ML) method in AMOS 24.0, with iterative fitting and refinement performed until satisfactory fit indices were achieved. The main fitting criteria are χ^2^/df<5, GFI>0.9, AGFI>0.9, RMSEA<0.05, where χ^2^/df is the chi-square degrees of freedom ratio, GFI is the goodness-of-fit index, AGFI is the adjusted goodness-of-fit index, RMSEA is the root-mean-square residual, SRMR is the standardized root-mean-square residual, CFI is the comparative goodness-of-fit index, NFI is the absolute fit index, and IFI is the absolute fit index. Fit index and IFI are the value-added fit index.

## 3 Results

### 3.1 Demographic characteristics

The survey respondents were 1,483 males (54.56%), with a male-to-female ratio of 1.201:1. The average age was 51.79 ± 10.20 years, mainly in the 45 to 60 age group, at 1429 (52.58%). Ethnicity was a predominantly Zhuang minority, totaling 2,562 (94.26%). Those in marriage were 2,303 (84.73%). The largest household size was 2 to 4 persons, 1,527 (56.18%). The highest number of people with an education level of junior high school or below was 2,479 (91.21%). Household registration was mainly local, with 2,695 persons (99.15%). Annual household income is mainly concentrated in 10,000 ~ 30,000 (RMB), 1,279 people (47.06%). Among the survey respondents, 588 people (21.63%) smoked daily, and 1,890 people (69.54%) never smoked. 1,642 people (60.41%) were of normal weight, and 139 people (5.11%) were obese in BMI. Only 780 (28.70%) participated in regular physical activity. Those who had heard of “HL66” were 977 (35.95%). Other details are shown in Table 1.

**Table 1:** Demographic characteristics of respondents (*N* = 2,718).

Feature grouping		*N*	%
Gender	Male	1,483	54.56
	Female	1,235	45.44
Age	15	57	2.10
	30	579	21.30
	45	1,429	52.58
	60	653	24.03
Ethnic	Han	156	5.74
	Zhuang and others	2,562	94.26
Marital status	Unmarried	187	6.88
	Married	2,303	84.73
	Separated/Divorced/Widowed	228	8.39
Family size	1	116	4.27
	2	1,527	56.18
	5	989	36.39
	8	86	3.16
Education	No/little literacy	155	5.70
	Primary school	1,091	40.14
	Junior high school	1,233	45.36
	Senior high school or above	239	8.79
Local household	Yes	2,695	99.15
	No	23	0.85
Income (K RMB)	<10	509	18.73
	10	1,279	47.06
	30	570	20.97
	50	360	13.25
Smoking	Smoke every day	588	21.63
	Smoke, not every day	62	2.28
	Former smoker	178	6.55
	Never smoke	1890	69.54
BMI	Underweight	279	10.26
	Normal weight	1,642	60.41
	Overweight	658	24.21
	Obese	139	5.11
Often physical exercise	Yes	780	28.70
	No	1938	71.30
“HL66”	Yes	977	35.95
	No	1741	64.05

### 3.2 Health literacy status and influencing factors

The total health literacy of farmers in the region was 18.21%, lower than the 21.62% of local urban and rural residents (results of the previous study) and even lower than the 25.00% of Guangxi residents (12) and the 27.78% of national residents (13) in the same period (*p* < 0.0083). All six categories of knowledge health literacy (HL01 - HL06) were lower than local, Guangxi, and national residents (*p* < 0.0083). See Table 2 for details.

**Table 2:** Health literacy level of farmers (%).

Item	1. Famer	2. Chongzuo	3. Guangxi	4. China	*χ*^2^-value	*p*-value	Pairwise *χ^2^*
Sample size	2,718	3,242	31,647	71,842	—	—	—
HL00/N	495	701	7,326	19,958	—	—	—
/%	18.21	21.62	23.15	27.78	369.802	0.000	1 < (2 = 3) < 4
HL01	33.08	37.54	45.24	53.55	1160.519	0.000	1 < 2 < (3 = 4)
HL02	21.27	24.52	25.54	28.16	137.449	0.000	1 < (2 = 3) < 4
HL03	22.41	25.82	28.58	28.85	65.330	0.000	1 < 2 < (3 = 4)
HL04	40.54	44.82	51.90	58.51	823.762	0.000	1 < 2 < 3 < 4
HL05	21.67	23.66	24.21	27.68	182.445	0.000	1 < (2 = 3) < 4
HL06	25.97	30.14	30.94	39.81	946.084	0.000	1 < (2 = 3) < 4

Adjusted test level α’ = 0.00833 for Pairwise χ^2^.

As seen in Table 3, health literacy is higher among males (19.89%) than females (16.19%). Health literacy tends to decrease with age. The health literacy of those whose marital status is “Separated/Divorced/Widowed” is only 11.84%, while that of the unmarried is the highest at 26.20%. The higher the population’s literacy level and annual household income, the higher their health literacy. The health literacy of “Never smoke” is the lowest at 17.14%. Health literacy was higher for regular exercise (21.15%) than infrequent exercise (17.03%). Health literacy was much higher among those who had heard of HL66 (25.90%) than those who had not (13.90%). The differences in health literacy among other demographic characteristics were not statistically significant (*p* > 0.05). See Table 3 for details.

**Table 3:** Results of univariate analysis of farmers’ health literacy and health outcomes n(%).

Feature grouping		HL	Self-assessment			Chronic	Accidental injury	Medical expenses (RMB)			
			Good	Common	Bad			0	1	1,000	5,000
Total		495 (18.21)	1,315 (48.38)	1,124 (41.35)	279 (10.26)	513 (18.87)	207 (7.62)	469 (17.26)	1,305 (48.01)	667 (24.54)	277 (10.19)
Gender	Male	295 (19.89)	785 (52.93)	572 (38.57)	126 (8.50)	299 (20.16)	112 (7.55)	296 (19.96)	720 (48.55)	330 (22.25)	137 (9.24)
	Female	200 (16.19)	530 (42.91)	552 (44.70)	153 (12.39)	214 (17.33)	95 (7.69)	173 (14.01)	585 (47.37)	337 (27.29)	140 (11.34)
	*χ*^2^-value	**6.186**	**30.039**			3.534	0.019	**23.900**			
	*p*-value	**0.013**	**0.000**			0.060	0.891	**0.000**			
Age	15	22 (38.60)	38 (66.67)	17 (29.82)	2 (3.51)	2 (3.51)	4 (7.02)	12 (21.05)	23 (40.35)	14 (24.56)	8 (14.04)
	30	193 (33.33)	349 (60.28)	210 (36.27)	20 (3.45)	38 (6.56)	26 (4.49)	115 (19.86)	255 (44.04)	155 (26.77)	54 (9.33)
	45	227 (15.89)	686 (48.01)	593 (41.05)	150 (10.05)	278 (19.45)	115 (8.05)	245 (17.14)	712 (49.83)	329 (23.02)	143 (10.01)
	60	53 (8.12)	242 (37.06)	304 (46.55)	107 (16.39)	195 (29.86)	62 (9.49)	97 (14.85)	315 (48.24)	169 (25.88)	72 (11.03)
	*χ*^2^-value	**154.659**	**100.551**			**117.905**	**12.785**	13.268			
	*p*-value	**0.000**	**0.000**			**0.000**	**0.005**	0.151			
Marital status	Unmarried	49 (26.20)	106 (56.68)	62 (33.16)	19 (10.16)	29 (15.51)	13 (6.95)	49 (26.20)	81 (43.32)	39 (20.86)	18 (9.63)
	Married	419 (18.19)	1,115 (48.42)	960 (41.68)	228 (9.90)	430 (18.67)	166 (7.21)	377 (16.37)	1,104 (47.94)	585 (25.40)	237 (10.29)
	Separated/Divorced/Widowed	27 (11.84)	94 (41.23)	102 (44.74)	32 (14.04)	54 (23.68)	28 (12.28)	43 (18.86)	120 (52.63)	43 (18.86)	22 (9.65)
	*χ*^2^-value	**14.229**	**83.921**			4.891	**7.713**	**16.814**			
	*p*-value	**0.001**	**0.000**			0.087	**0.021**	**0.010**			
Education	No/little literacy	6 (3.87)	47 (30.32)	79 (50.97)	29 (18.71)	44 (28.39)	19 (12.26)	13 (8.39)	81 (52.26)	41 (26.45)	20 (12.90)
	Primary school	131 (12.01)	470 (43.08)	479 (43.90)	142 (13.02)	218 (19.98)	77 (7.06)	185 (16.96)	533 (48.85)	259 (23.74)	114 (10.45)
	Junior high school	280 (22.71)	666 (54.01)	471 (38.20)	96 (7.79)	207 (16.79)	95 (7.70)	235 (19.06)	584 (47.36)	302 (24.49)	112 (9.08)
	Senior high school or above	78 (32.64)	132 (55.23)	95 (39.75)	12 (5.02)	44 (18.41)	16 (6.69)	36 (15.06)	107 (44.77)	65 (27.20)	31 (12.97)
	*χ*^2^-value	**99.721**	**68.082**			**13.572**	5.533	**17.176**			
	*p*-value	**0.000**	**0.000**			**0.004**	0.137	**0.046**			
Income (K RMB)	<10	57 (11.20)	200 (39.29)	223 (43.81)	86 (16.90)	126 (24.75)	46 (9.04)	77 (15.13)	268 (52.65)	117 (22.99)	47 (9.23)
	10	224 (17.51)	604 (47.22)	553 (43.24)	122 (9.54)	248 (19.39)	85 (6.65)	218 (17.04)	593 (46.36)	331 (25.88)	137 (10.71)
	30	115 (20.18)	304 (53.33)	218 (38.25)	48 (8.42)	96 (16.84)	53 (9.30)	110 (19.30)	272 (47.72)	142 (24.91)	46 (8.07)
	50	99 (27.50)	207 (57.50)	130 (36.11)	23 (6.39)	43 (11.94)	23 (6.39)	64 (17.78)	172 (47.78)	77 (21.39)	47 (13.06)
	*χ*^2^-value	**39.553**	**53.302**			**24.544**	6.236	14.961			
	*p*-value	**0.000**	**0.000**			**0.000**	0.101	0.092			
Smoking	Smoke every day	114 (19.39)	348 (59.18)	197 (33.50)	43 (7.31)	82 (13.95)	48 (8.16)	125 (21.26)	293 (49.83)	129 (21.94)	41 (6.97)
	Smoke, not every day	18 (29.03)	43 (69.35)	14 (22.58)	5 (8.06)	7 (11.29)	6 (9.68)	19 (30.65)	28 (45.16)	13 (20.97)	2 (3.23)
	Former smoker	39 (21.91)	82 (46.07)	78 (43.82)	18 (10.11)	47 (26.40)	22 (12.36)	33 (18.54)	72 (40.45)	44 (24.72)	29 (16.29)
	Never smoke	324 (17.14)	842 (44.55)	835 (44.18)	213 (11.27)	377 (19.95)	131 (6.93)	292 (15.45)	912 (48.25)	481 (25.45)	205 (10.85)
	*χ*^2^-value	**8.504**	**50.856**			**19.670**	6.746	**37.106**			
	*p*-value	**0.037**	**0.000**			**0.000**	0.080	**0.000**			
BMI	Underweight	43 (15.41)	106 (37.99)	124 (44.44)	49 (17.56)	56 (20.07)	27 (9.68)	42 (15.05)	118 (42.29)	82 (29.39)	37 (13.26)
	Normal weight	296 (18.03)	820 (49.94)	668 (40.68)	154 (9.38)	260 (15.83)	134 (8.16)	302 (18.39)	795 (48.42)	395 (24.06)	150 (9.14)
	Overweight	126 (19.15)	316 (48.02)	279 (42.40)	63 (9.57)	152 (23.10)	37 (5.62)	106 (16.11)	322 (48.94)	160 (24.32)	70 (10.64)
	Obese	30 (21.58)	73 (52.52)	53 (38.13)	13 (9.35)	45 (32.37)	9 (6.47)	19 (13.67)	70 (50.36)	30 (21.58)	20 (14.39)
	*χ*^2^-value	2.954	**25.053**			**34.390**	6.349	16.033			
	*p*-value	0.399	**0.000**			**0.000**	0.096	0.066			
Physical exercise	Yes	165 (21.15)	436 (55.90)	279 (35.77)	65 (8.33)	142 (18.21)	67 (8.59)	140 (17.95)	384 (49.23)	184 (23.59)	72 (9.23)
	No	330 (17.03)	879 (45.36)	845 (43.60)	214 (11.04)	371 (19.14)	140 (7.22)	329 (16.98)	921 (47.52)	483 (24.92)	205 (10.58)
	*χ*^2^-value	**6.356**	**25.000**			0.320	1.475	2.036			
	*p*-value	**0.012**	**0.000**			0.572	0.225	0.566			
“HL66”	Yes	253 (25.90)	580 (59.37)	341 (34.90)	56 (5.73)	139 (14.23)	60 (6.14)	192 (19.65)	487 (49.85)	202 (20.68)	96 (9.83)
	No	242 (13.90)	735 (42.22)	783 (44.97)	223 (12.81)	374 (21.48)	147 (8.44)	277 (15.91)	818 (46.98)	465 (26.71)	181 (10.40)
	*χ*^2^-value	**60.456**	**83.921**			**21.511**	**4.714**	**15.627**			
	*p*-value	**0.000**	**0.000**			**0.000**	**0.030**	**0.001**			

This statistical table is not presented because the differences are not statistically significant when comparing the variables by ethnicity, family size, and household registration in “Feature grouping”.

Bold values indicate statistically significant findings.

### 3.3 Health outcome status and influencing factors

#### 3.3.1 Health self-assessment

Health self-assessment was good, fair, and poor, with 48.38, 41.35, and 10.26%, respectively. Self-assessed health status was better in males than females. As age increases, the self-assessed health status gets worse. 6.96% of the respondents under 45 years old were “bad,” 10.05% in the 45–60 age group, and 16.39% in the 65–69 age group and above. The marital status of “Separated/Divorced/Widowed” was rated as “bad” by 14.04% of the respondents, which is higher than that of “Unmarried” and “Married.” This is higher than the 9.92% (247/2490) for “Unmarried” and “Married.” The higher the level of education, the better the self-assessment status, with 54.21% (798/1472) assessing themselves as good at junior high school and above, compared to 41.49% (798/1472) at junior high school and below. Similarly, the higher the annual household income, the better the self-rated status. The health self-assessment status of those who smoked (daily or occasional) was 60.15% (391/650), higher than that of non-smokers (including quitters), which was 44.68% (924/2068). Regarding BMI, the health self-assessment status of “Obese” was higher for those who rated themselves as good (52.52%), while the self-assessment status of “Poor” was “Underweight” was higher (17.56%). The health self-assessment status was better for those who were physically active and those who had heard of “HL66.” See Table 3 for details.

#### 3.3.2 Chronic diseases

A total of 513 people (18.87%) suffered from chronic diseases, of whom 51 (1.88%) suffered from two or more chronic diseases. The classification of chronic diseases is shown in Table 4, with the higher incidence of Hypertension, Diabetes, and Heart disease in that order. It is worth noting that of the 135 cases of “Else” (23.64%), 34 (5.95%) were gastric disorders and 31 (5.43%) were cervical, lumbar, joint and bone disorders.

**Table 4:** Types of chronic disease and accidental injury by farmers.

Type of health outcomes		*N*	Composition ratio (%)	Prevalence rate (‰)
Chronic disease	Hypertension	305	53.42	112.21
	Heart disease	35	6.13	12.88
	Cerebrovascular diseases	30	5.25	11.04
	Diabetes	55	9.63	20.24
	Malignant tumors	11	1.93	4.05
	Else	135	23.64	49.67
	Total	571	100.00	—
Accidental injury	Motor Vehicle Car Accident	15	6.05	5.52
	Non-Motorized Vehicle Accidents	14	5.65	5.15
	Falls	102	41.13	37.53
	Blunt Force Injury	16	6.45	5.89
	Firearm Injury	5	2.02	1.84
	Knife/Sharp Object Injuries	55	22.18	20.24
	Burns	26	10.48	9.57
	Poisoning	3	1.21	1.10
	Animal Injuries	5	2.02	1.84
	Other	7	2.82	2.58
	Total		100.00	—

Because the number of cases of “Suffocation/Hanging”, “Drowning”, and “Sexual assault” in Accidental Injury is all zero, they are not presented in this statistical table.

Table 3 shows that the prevalence of chronic diseases increases significantly with age. The education level of elementary school and below accounted for 51.07% (262/513) of chronic diseases, and the prevalence rate of 21.03% (262/1246) was the highest. The higher the household’s annual income, the lower the percentage of chronic diseases. The highest percentage of chronic diseases was found in the “Former smoker” group (26.40%), while the highest BMI was found in the “Obese” group (32.37%) and the lowest in the “Normal weight” group (262/1246). For BMI, “Obese” had the highest (32.37%) and “Normal weight” the lowest (15.83%). The percentage of chronic diseases that had heard of “HL66” was significantly lower at 14.23% than at 21.48% for those who had not heard of it. Other characteristics, such as gender, marital status, and regular physical activity, were not statistically significant. See Table 3 for details.

#### 3.3.3 Accidental injuries

A total of 207 (7.62%) had suffered accidental injuries in the past year, of which 30 (1.10%) had suffered two or more. The classification of unintentional injuries is shown in Table 4. The incidence in descending order is Falls, Knife/Sharp Object Injuries, Burns, Blunt Force Injuries, and Motor Vehicle Car accidents.

Table 3 shows that the lowest incidence (4.49%) was found in the age group of 30–45 years, and the incidence tended to increase with decreasing and increasing age. The highest incidence rate (12.28%) was found in the group with marital status “Separated/Divorced/Widowed,” and the same was found in the group with literacy level “No/little literacy” (12.26%). The prevalence of having heard of “HL66” was significantly lower in 6.14% than in 8.44% of those who had not heard of it. The differences in the occurrence of unintentional injuries for the other characteristics were not statistically significant. See Table 3 for detail.

#### 3.3.4 Out-of-pocket medical expenses

A total of 469 people (17.26%) paid “0” out-of-pocket medical expenses in the past year, and 65.27% (1774/2718) paid <1,000 RMB, median = 300 RMB (P5 = 0, P95 = 9,050). Table 3 shows statistically significant differences in out-of-pocket costs by gender, marital status, education, smoking status, and having heard of “HL66.” It was higher for females than for males and higher for those who were married. No/little literacy” and ‘Senior high school or above’, i.e., low education and high education, had a larger proportion of higher medical expenses. The “Never smoke” group had a larger proportion of higher costs. Those who had not heard of “HL66” had higher expenses.

### 3.4 Correlation of demographic characteristics, health literacy, and health outcomes

The results of the correlation analysis showed that the age (r = −0.229, *p* < 0.01), marital status (r = −0.072, *p* < 0.01), education level (r = 0.190, *p* < 0.01), income (r = 0.115, *p* < 0.01), and whether or not the local farmers had heard of “HL66 “(r = −0.149, *p* < 0.01) were all associated with health literacy. Meanwhile, health literacy was associated with all indicators of health outcomes other than unintentional injuries. There was a positive correlation between all indicators of health outcomes. See Table 5 for details.

**Table 5:** Demographic characteristics, health literacy, and health outcome correlates.

Variables	1	2	3	4	5	6	7	8	9	10
1. Age	1.000									
2. Marital status	**0.249** ^**^	1.000								
3. Education	**−0.151** ^**^	**−0.108** ^**^	1.000							
4. Incomes	**−0.155** ^**^	−0.032	**0.149** ^**^	1.000						
5. HL66	**0.039** ^*^	−0.008	**−0.149** ^**^	**−0.103** ^**^	1.000					
6. HL00	**−0.229** ^**^	**−0.072** ^**^	**0.190** ^**^	**0.115** ^**^	**−0.149** ^**^	1.000				
7. Self-assessment	**0.186** ^**^	**0.060** ^**^	**−0.148** ^**^	**−0.127** ^**^	**0.175** ^**^	**−0.098** ^**^	1.000			
8. Chronic	**0.207** ^**^	**0.042** ^*^	**−0.054** ^**^	**−0.092** ^**^	**0.089** ^**^	**−0.081** ^**^	**0.296** ^**^	1.000		
9. Accidental injury	**0.061** ^**^	**0.042** ^*^	−0.014	−0.007	**0.042** ^*^	**−0.053** ^**^	**0.116** ^**^	**0.074** ^**^	1.000	
10. Medical expenses	0.027	0.006	−0.015	−0.011	**0.066** ^**^	−0.007	**0.276** ^**^	**0.239** ^**^	**0.160** ^**^	1.000

*p<0.05, **p<0.01.

Bold values indicate statistically significant findings.

### 3.5 Structural equation modeling

**Figure 1:**
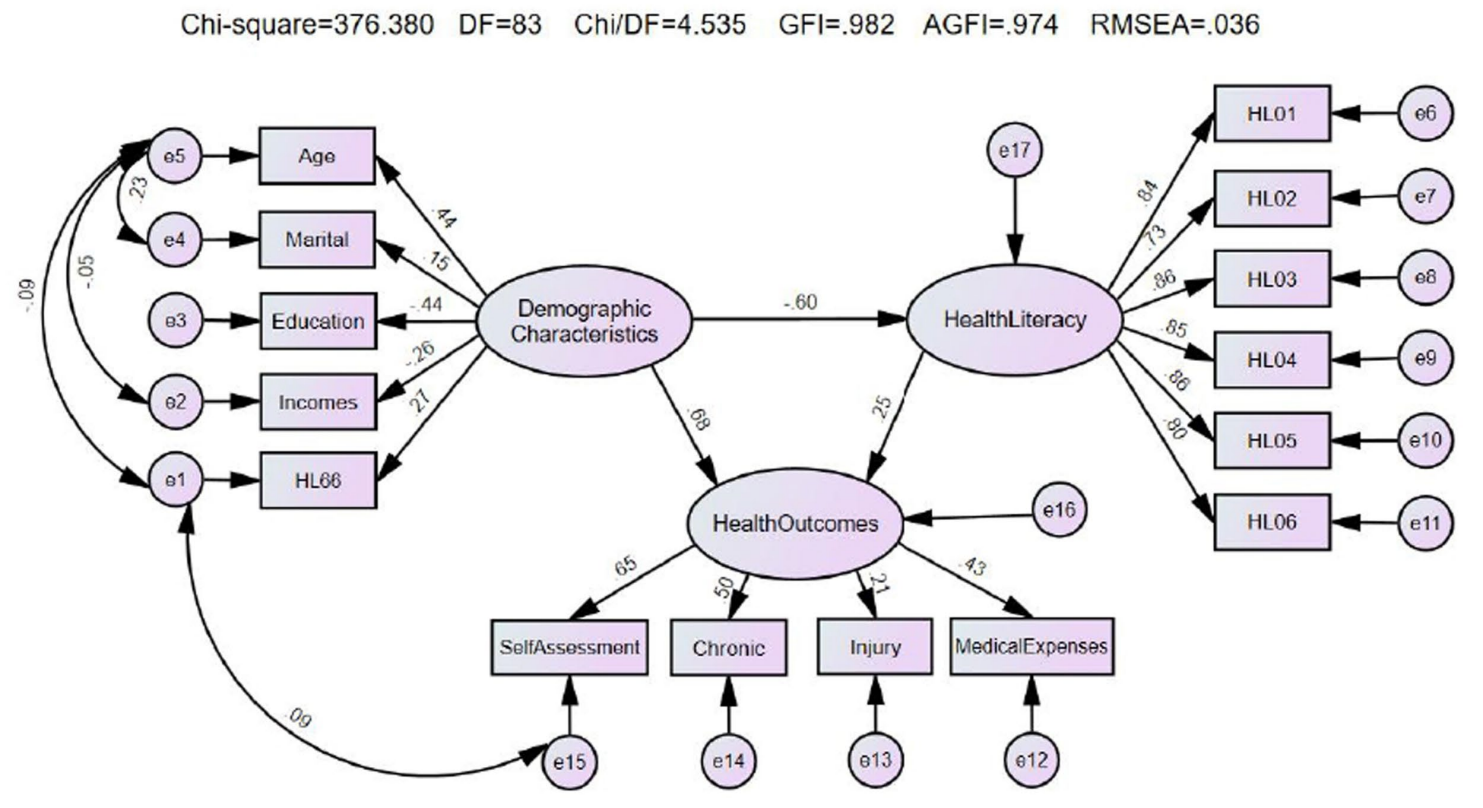
Diagram of the modified fitting pattern for the structural equation modeling: the three latent variables were respectively: demographic characteristics, health literacy, and health outcomes. The exogenous variables of demographic characteristics were age, marital status, educational level, annual household income, and “HL66.” The exogenous variables of health literacy (HL00) included the six categories of knowledge health literacy, HL01–HL06. And the health outcomes were measured by the four exogenous variables of self-assessment of health, chronic diseases, accidental injuries, and out-of-pocket medical expenses. The values on the arrow lines were represented standardized path factor.

The constructed structural equation model was fitted by deleting the paths with insignificant path coefficients and correcting and fitting the correlations several times according to the M. I.’s prompts until each fitting index reached the standard. See Table 6. The corrected structural equation model is shown in Figure 1.

**Table 6:** Modified model fit indices.

Fitness index	χ^2^/df	RMSEA	SRMR	GFI	AGFI	CFI	NFI	IFI
Fitting Criteria	< 5	< 0.05	< 0.05	> 0.9	> 0.9	> 0.9	> 0.9	> 0.9
Revised model	4.535	0.036	0.031	0.982	0.974	0.979	0.973	0.979

The path coefficients between the latent variables in the model show that all exogenous latent variables significantly affect endogenous latent variables. The standardized coefficients of all the pathways are *p* < 0.001, which reaches a significant level, indicating that the path is established and that the influencing factors among the pathways have an interaction relationship. See Table 7 for details.

**Table 7:** Validated factor analysis of the modified model (*p*-value).

Path	Standardized path factor	Path factor	*S. E.*	*C. R*	*p*
Health Literacy ← Demographic Characteristics	−0.603	−12.479	1.504	−8.295	<0.001
Health Outcomes ← Demographic Characteristics	0.682	1.968	0.323	6.089	<0.001
Health Outcomes ← Health Literacy	0.246	0.034	0.009	3.986	<0.001
HL66 ← Demographic Characteristics	0.269	1.000			
Incomes ← Demographic Characteristics	−0.260	−1.854	0.259	−7.165	<0.001
Education ← Demographic Characteristics	−0.438	−2.482	0.282	−8.798	<0.001
Marital ← Demographic Characteristics	0.146	0.442	0.089	4.936	<0.001
Age ← Demographic Characteristics	0.440	2.501	0.289	8.656	<0.001
HL01 ← Health Literacy	0.843	1.000			
HL02 ← Health Literacy	0.729	0.521	0.012	43.869	<0.001
HL03 ← Health Literacy	0.862	1.064	0.019	56.708	<0.001
HL04 ← Health Literacy	0.854	1.280	0.023	55.837	<0.001
HL05 ← Health Literacy	0.855	1.112	0.020	56.042	<0.001
HL06 ← Health Literacy	0.797	0.688	0.014	50.035	<0.001
Medical Expenses ← Health Outcomes	0.430	1.000			
Injury ← Health Outcomes	0.206	0.147	0.019	7.525	<0.001
Chronic ← Health Outcomes	0.496	0.522	0.040	13.173	<0.001
Self-Assessment ← Health Outcomes	0.654	1.165	0.090	12.877	<0.001

Demographic characteristics significantly affected health literacy with a total effect of −0.603, a direct effect of −0.603, and no indirect effect. Health literacy also significantly affected health outcomes, with a total effect of 0.246, a direct effect of 0.246, and no indirect effect. Demographic characteristics significantly affected health outcomes, with a total effect of 0.534, a direct effect of 0.682, and an indirect effect of −0.148. See Table 8.

**Table 8:** Effect relationships between potential variables.

Relationship of latent variables	Total effect	Direct effect	Indirect effect
Demographic Characteristics → Health Literacy	−0.603	−0.603	—
Health Literacy → Health Outcomes	0.246	0.246	—
Demographic Characteristics → Health Outcomes	0.534	0.682	−0.148

## 4 Discussion

Farmers, as a broad and important population, directly affect food security, economic development, and social stability. USDA (United States Department of Agriculture) data show that the total number of people working on farms globally in 2020 will be about 841 million (17), accounting for more than a quarter of the global labor force. China’s rural population is 499,814,600 people in 2023, accounting for about 35.43% (18). In Guangxi, at the end of 2023, the region’s resident population was 50.27 million, and the rural population was 21.725 million, accounting for 43.22% (19). Chongzuo City is located in the southwest of Guangxi, under the jurisdiction of seven administrative districts, four of which border with Vietnam. The population in rural areas is 1,170,900, accounting for 56.06% of the total population (20). The sample of this study accounted for 83.84% of the monitored population. In the context of global health equity and the implementation of the Healthy China strategy, farmers are a group that cannot be ignored in health education and health promotion, especially in remote border areas.

The results of the study found that the total health literacy of farmers in the border areas of South China was 18.21%, which was lower than the average level of the residents of the country, Guangxi and Chongzuo, during the same period, and the same for all dimensions of health literacy. However, when compared with various groups of farmers in multiple regions, for example, the health literacy of farmers in Zhejiang Province was 34.32% (21), 19.84% in Henan Province (22), 17.50% in Shiyan City, Hubei Province (23), 16.2% of Bai rural residents in Dali Region (24), 6.5% in Liangshan Prefecture, Sichuan Province (25), and Uyghur farmers in Kashgar Region, Xinjiang Province 3.93% (26). The health literacy of local farmers was lower than that of farmers in developed regions and comparable to that of central China but higher than that of farmers in western regions, especially in ethnic minority areas.

As far as health outcomes are concerned. First, health self-assessment, 48.38% of the subjects in this study had good health self-assessments. The proportion of farmers in the Changshou District of Chongqing was 39.5% (27), 58.59% of rural residents in Xuzhou, Jiangsu Province, considered their health to be good (28), and up to 66.8% of rural residents of the Bai ethnic group in the Dali area (24). This shows that the health literacy of farmers on the South China border is low, but the health self-assessment status should be in the middle level of the country. Second, chronic diseases, suffering from chronic diseases accounted for 18.87%, lower than 29.98% of Zhejiang residents (29) and 24.61% of Hebei Province residents (30) during the same period, slightly higher than the 17.52% of local urban and rural residents in Chongzuo (the results of our team’s previous study) and the 17.7% of Bai rural residents in the Dali area (24), and significantly higher than the 16.29% of residents in Gansu Province (31) and the 9.23% of Xinjiang Kashgar region residents’ 9.23% (32). Combining the differences between east and west China and the urban–rural differences, the proportion of farmers suffering from chronic diseases in this city is in the middle to lower level in China. Third, unintentional injuries, the prevalence of unintentional injuries among local farmers was 7.62%. An epidemiologic survey of unintentional injuries in Shandong Province, China, for rural residents had an annual injury incidence rate of 7.79% (33). A study showed that the standardized mortality rate of unintentional injuries in the Chinese population from 2005 to 2021 was mainly concentrated in the age group of 15–44 years. The standardized mortality rate of unintentional injuries in the western region was higher than that in the eastern and central regions.

In comparison, the standardized mortality rate of unintentional injuries in the rural population was higher than that in the city. The standardized mortality rate of unintentional injuries in the urban and rural populations was on a decreasing trend (34). Another study based on the China Cause of Death Surveillance Dataset (CCDS) identified male, rural, and western residents as the key populations to prevent unintentional injuries from occurring (35). When the incidence of unintentional injuries among farmers is not low, health education efforts must focus on this program. In addition, Chongzuo City is known as the “Sugar Capital of China”, which is the largest sugarcane planting base and sugar production base in China, and the local farmers’ “Knife/Sharp Object Injuries” and “Blunt Force Injuries” are the most common injuries among farmers. “Knife/Sharp Object Injuries” and “Blunt Force Injuries” of local farmers have a lot to do with the nature of their work. Fourth, out-of-pocket medical expenses, the median out-of-pocket medical expenses of local farmers in the past year was 300 RMB. 1632.47 RMB per capita healthcare expenditure in rural areas in 2022, according to the National Bureau of Statistics (36), and 20,133 RMB per capita disposable income in rural areas in 2022 (37), suggesting that the out-of-pocket medical expenses of the study population are still low.

Stratified analysis revealed statistically significant differences in health literacy by gender, age, marital status, education, income, smoking, physical exercise, and HL66, suggesting these factors are correlates of farmers’ health literacy in the study region. Consistent with prior studies in Denmark (8), Portugal (38), and central China (39), health literacy was significantly associated with demographic characteristics such as gender, age, education, and income. Demographic characteristics differentially influence health outcomes. Traditional factors—age, education, and household income—affect self-assessed health, chronic diseases, unintentional injuries, and out-of-pocket medical expenses, corroborating previous research (40–42). Marital status significantly impacts health and mortality (41, 43); individuals in less happy marriages report poorer health (43). The present study found that farmers who were separated/divorced/widowed exhibited significantly adverse health outcomes across three of four measures (excluding chronic diseases, *p* = 0.087), highlighting the vulnerability of this group. HL66—the 66 Articles of Health Literacy for Chinese Citizens, issued by the NHC in 2008 (44), and promoted nationwide (45) demonstrated a significant positive effect on all four health outcomes. This guideline has proven effective in improving health literacy among the general population, and our findings indicate that such benefits extend to border farmers, enhancing both health literacy and health outcomes.

This study is the first to take farmers in border areas as the research object and construct structural equation modeling to explore the effect relationship between demographic characteristics, health literacy, and health outcomes. Previous similar studies focused on the relationship between socioeconomic status (occupation, education, income, etc.) and health literacy (46). Some studies considered health outcomes as a potential variable, but most included observational variables: health self-assessment and chronic diseases (24, 47–49). In the present study, occupation was excluded from demographic characteristics and marital status, and “HL66,” which is rarely reported in the literature, was included. In addition, the rarely reported unintentional injuries and medical expenses were included in the health outcome variables. The results showed that the model fit the data well, and all paths were statistically significant (P0.05). Health outcomes were significantly associated with both demographic characteristics and health literacy, while the level of health literacy was confirmed as a major predictor of health outcomes. Demographic characteristics were significantly and negatively correlated with knowledge of the six categories of health literacy. Pathways indicated that the older the age, the lower the education level, the lower the annual household income, the marital status of “Separated/Divorced/Widowed” compared to unmarried and married, and not having heard of “HL66,” the lower the level of total health literacy. The level was lower. Demographic characteristics were significantly positively associated with health outcomes. Pathways, education level, and annual household income were protective factors for health outcomes. Increasing age, marital status of “Separated/Divorced/Widowed” and not having heard of “HL66” were unfavorable factors for health outcomes. Health literacy is a protective factor for health outcomes. That is, the higher the level of health literacy, the better the self-assessed health status, the lower the prevalence of chronic diseases and unintentional injuries, and the lower the out-of-pocket medical expenses.

Based on these results, border farmers’ health education and health promotion should focus on subgroups over 45 years old, elementary school education or less, Separated/Divorced/Widowed marital status, and annual household income less than 50 K RMB. Improving the health literacy of border farmers can improve their health outcomes, reduce the incidence of chronic diseases and unintentional injuries, and reduce medical expenses. When implementing health education, primary health care workers, social workers, or volunteers should make improving health literacy one of their core tasks. In promoting health education from a broader and deeper perspective, the promotion of “HL66” is an effective measure that can be adapted to local conditions.

## 5 Limitations

There are two limitations of this study. One is that this study focuses on farmers in only one prefecture-level city on the border of South China, affecting the findings’ generalizability. The second is that the study results were derived from a cross-sectional sample, thus failing to highlight the direction of causality of certain observed factors. To solve these problems, prospective cohort studies can be used. Two, the model could be validated using multiple samples focusing on farmers in other parts of South China and Southwest China to validate the findings further.

## 6 Conclusion

The health literacy level of farmers in the border areas of South China is low compared to the national level and the level of their provinces, and it is also relatively low compared to the rural areas of developed provinces. Health outcomes are moderate, and out-of-pocket medical expenses are low. Health literacy is directly affected by age, marital status, education level, income, and other demographic characteristics, and farmers’ health self-assessment, chronic diseases, accidental injuries, and out-of-pocket medical expenses are directly affected by health literacy and indirectly by demographic characteristics. Middle-aged and older adults farmers with Separated/Divorced/Widowed marital status, low education level, and low income are the key populations for health education and health promotion. The “HL66” in health education is an effective measure that should be further promoted and strengthened. Farmers’ health literacy levels can be raised, and health outcomes can be improved by tailoring health education or promotion efforts to local conditions.

## Data Availability

The original contributions presented in the study are included in the article/supplementary material, further inquiries can be directed to the corresponding authors.
